# Temperature dependency of immunoglobulin production in novel human partner cell line

**DOI:** 10.1186/1753-6561-7-S6-P37

**Published:** 2013-12-04

**Authors:** Galina Kaseko, Marjorie Liu, Edwin Hoe, Qiong Li, Mercedes Ballesteros, Tohsak Mahaworasilpa

**Affiliations:** 1 The Stephen Sanig Research Institute, Sydney, NSW, 2015 Australia

## Introduction

A number of immunoglobulin (Ig) secreting human hybrid cell lines were created using one-on-one somatic cell hybridization of a rare human tumor infiltrating B lymphocyte and a cell of a novel human cell line (WTM), developed in house and described earlier [1]. These hybrid cell lines secret various amounts of tumor-derived immunoglobulins (Igs) of different specificities. Current investigative efforts are directed towards determining the optimal culture conditions to ensure consistent cell growth and long-term stabilities of Ig productions by the hybrids. Based on previous literature reports [2,3], we investigated an effect of short- and long-term mild hypothermic conditions on Ig production, cell growth and cell size.

## Results

Three different hybrid cell lines each representing the highest, medium and lowest ranges of Ig productions, were subject to culture temperature drops from 37°C to 36°C, 35°C or 34°C for up to 168 hours with 24-hour data point intervals. In case of prolonged mild hypothermia, the cell line with Ig production most susceptible to temperature drops was maintained at various temperatures below 37°C (e.g. 36°C, 35°C and 34°C) for at least 5 passages with each passage lasting 120 hours and the data taken at a 24-hour interval. At each data point for each of the hybrid cell lines at a given temperature interval, the sample was collected to determine cell concentration, cell size and Ig production.

**Figure 1:**
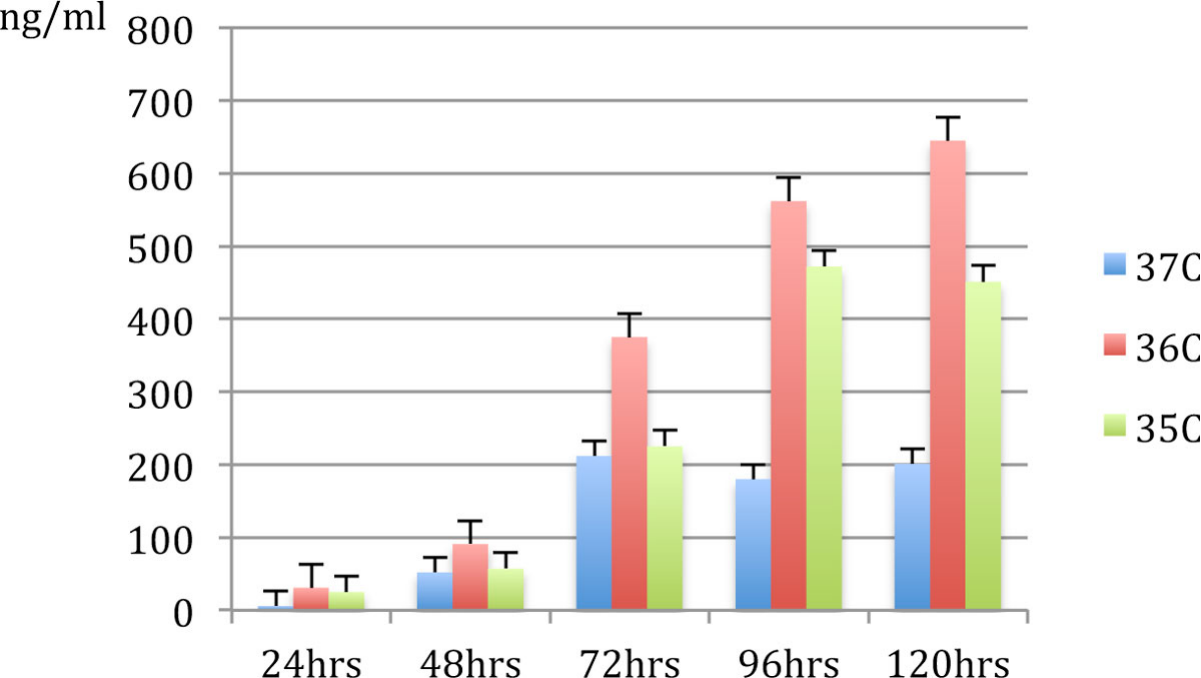
**Effects of short-term temperature drops from 37°C to 36°C and 37°C to 35°C on Ig production by hybrid cell line 2**.

Whilst there was no observable effect of any of the short-term temperature drops on the cell growth or the cell size in any of the three hybrid cell lines, the level of Ig concentration consistently increased in all of them, with gains ranging from 67% and 320% and with Ig productivity peaking between 48 and 72 hours after the exposure to lower temperatures (Figure 1).

In contrast to short-temperature drop conditions, a prolonged exposure to mild hypothermic conditions (longer than 1 passage) led to a progressive decrease in cell size over 5 passages. This decrease in the cell size was accompanied by gradual 10-30% gains of Ig production with each passage after the initial 100 to 150% increase in Ig concentration immediately upon transfer to lower temperature (Table 1). When cultured at 36°C, it seems to generate the highest increase in Ig production. This temperature effect was not noticeable at log phase of cell growth.

**Table 1:** Effects of prolonged mild hypothermia on Ig production by hybrid cell line 2 at day 5 of each passage over 5 passages.

Passage	P0 (ng/ml)	P1 (ng/ml)	P2 (ng/ml)	P3 (ng/ml)	P4 (ng/ml)	P5 (ng/ml)
37°C	342	322	388	356	301	348
36°C	0	712	758	929	1559	928
35°C	0	605	452	514	586	716
34°C	0	751	667	535	490	465

## Conclusions

In conclusion, whilst lowering temperature in the culture resulted in overall increase in Ig concentration, our results suggest that there might be different mechanisms responsible for the increase in Ig productivity in response to short temperature drop and prolonged hypothermia.
